# Shrunken methodology to genome-wide SNPs selection and construction of SNPs networks

**DOI:** 10.1186/1752-0509-4-S2-S5

**Published:** 2010-09-13

**Authors:** Yang Liu, Michael Ng

**Affiliations:** 1 Centre for Mathematical Imaging and Vision, and Department of Mathematics, Hong Kong Baptist University, Hong Kong

## Abstract

**Background:**

Recent development of high-resolution single nucleotide polymorphism (SNP) arrays allows detailed assessment of genome-wide human genome variations. There is increasing recognition of the importance of SNPs for medicine and developmental biology. However, SNP data set typically has a large number of SNPs (e.g., 400 thousand SNPs in genome-wide Parkinson disease data set) and a few hundred of samples. Conventional classification methods may not be effective when applied to such genome-wide SNP data.

**Results:**

In this paper, we use shrunken dissimilarity measure to analyze and select relevant SNPs for classification problems. Examples of HapMap data and Parkinson disease (PD) data are given to demonstrate the effectiveness of the proposed method, and illustrate it has a potential to become a useful analysis tool for SNP data sets. We use Parkinson disease data as an example, and perform a whole genome analysis. For the 367440 SNPs with less than 1% missing percentage from all 22 chromosomes, we can select 357 SNPs from this data set. For the unique genes that those SNPs are located in, a gene-gene similarity value is computed using GOSemSim and gene pairs that has a similarity value being greater than a threshold are selected to construct several groups of genes. For the SNPs that involved in these groups of genes, a statistical software PLINK is employed to compute the pair-wise SNP-SNP interactions, and SNPs with significance of *P* < 0.01 are chosen to identify SNPs networks based on their *P* values. Here SNPs networks are constructed based on Gene Ontology knowledge, and therefore each SNP network plays a role in the biological process. An analysis shows that such networks have relationships directly or indirectly to Parkinson disease.

**Conclusions:**

Experimental results show that our approach is suitable to handle genetic variations, and provide useful knowledge in a genome-wide SNP study.

## Background

Single Nucleotide Polymorphism (SNP) is a DNA sequence variation occurring when a single nucleotide - A, C, G, or T - differs at the same position between individuals [1]. SNPs are believed to result in differences between individuals, such as susceptibility to diseases [2]. They are abundant in human genome [3,4], which are considered as invaluable markers and potential powerful tools for both of genetic researches and applications in practice [5-8], for instance, disease gene discovery [9], drug development [10], and clinical treatment [11]. It is believed that more and more genetic researches and practical applications combined with machine learning or statistical methods will be investigated based on SNP data sets as SNPs will provide more useful information which is not shown by other methods.

In a SNP data set, the association between a disease and a set of relevant SNPs are investigated. Patients and normals are often categorized in groups according to their SNP genotypes (categorical values). Thousands of SNPs in different regions of chromosomes are used to describe characteristics of patient/normal samples. There are two key properties of data sets for such classification task: high-dimensional and categorical.

When many SNPs are used to detect the association between a disease and multiple marker genotypes, it is common to find only several numbers of SNPs having genotype patterns that are highly specific to each group of individuals. The SNPs are called the relevant SNPs, as opposed to the irrelevant SNPs that do not help much in identifying the group (i.e., individuals of the same type). Due to the large number of SNPs being irrelevant to each group, two individuals in the same group could have low similarity when measured by a simple similarity function that consider the genotypes of all SNPs. The groups may thus be undetectable by classification algorithms.

Many researchers gave efforts to find such a cohort of SNPs that having genotype patterns and highly specific to each group of individuals. Dai et al. [12] proposed a SNP-Haplotype Adaptive Regression (SHARE) algorithm that seeks the most informative set of SNPs for genetic association in a targeted candidate region by growing and shrinking haplotypes with one more or less SNP in a stepwise fashion, and comparing prediction errors of different models via cross-validation. Xu et al. [13] developed a set of web-based SNP selection tools which can select SNPs based on Genome-wide Association Studies (GWAS) results, linkage disequilibrium (LD), and predicted functional characteristics of both coding and non-coding SNPs. An example using prostate cancer was demonstrated that it can select a small panel of SNPs that include many of the recently validated prostate cancer SNPs. Latourelle et al. [14] sought to identify onset age genetic modifiers using genome-wide association study in familial Parkinson disease (PD). Meta analysis across three studies detected consistent association (*P* < 10^−5^) of five SNPs suggesting an influence of genes involved in endocytosis and lysosomal sorting in PD pathogenesis. Gao et al. [15] conducted a genome-wide parametric and nonparametric linkage analysis and found two loci for PD, indicating that additional PD susceptibility genes might be identified through targeted candidate gene studies in these loci regions. Srinivasan et al. [16] considered pathway association of SNP variation, which may have inconsistencies with traditionally individual SNP associations, providing a combination of the pathway and SNP analysis in the future.

The classification problem is defined for such a scenario, see for instance [17]. Each group is a set of individuals with an associated set of relevant SNPs such that in the group formed by the relevant SNPs, the individuals are similar to each other but dissimilar to individuals outside the group. In this paper, we test the HapMap data which is downloaded from HapMap webpage [18] and Parkinson disease genome-wide SNPs genotyping data obtained from the Coriell Institute for Medical Research. A new computational method called the nearest shrunken centroid was performed to select SNPs from these two data sets. In the literature, Schwender [19] has developed SAM for analysis of SNP data. The method is to study contingency table for testing if the distribution of the genotypes of SNPs differs between different groups. The Pearson *χ*^2^ statistic is used to handle rejection hypothesis. Shrunken *χ*^2^ statistics are further constructed to analyze relevant SNPs. In [20], Park et al. have considered using a classical nearest shrunken centroid method [21,22] to select SNPs. Their idea is to represent genotypes by numerical numbers directly and then perform the nearest shrunken centroid on the numerical data set of genotypes. The classical nearest shrunken centroid method is used to handle numerical microarray data sets. The main aim of this paper is to apply a new nearest shrunken centroid method to handle SNPs data in a categorical manner, and detect association between a disease and multiple marker genotypes based on a set of relevant SNPs selected. In addition, we conduct a comparison between our method and Park's [20] method based on one of the chromosomes. Genes that those selected SNPs located in are constructed several groups of genes using GOSemSim [23] with a similarity value being greater than a threshold. SNPs involved in these networks were further checked pair-wise SNP-SNP interactions using PLINK [24] with statistical significance of *P* < 0.01, which can be considered as an extension of existing Gene Ontology [25] knowledge.

## Methods

### Data source

#### *HapMap data*

The HapMap SNPs data [18] are downloaded from the HapMap webpage. According to the LD map of chromosome 22, see [26], 200 SNPs from chromosome 22 of 4 populations: Utah residents with ancestry from northern and western Europe (CEU), Han Chinese in Beijing, China, (CHB), Japanese in Tokyo, Japan (JPT) and Yoruba in Ibadan, Nigeria (YRI) are picked out randomly from a region from 3.44e7−3.5e7 kb [27], which shows a great difference of SNP positions on the LD map over 4 populations. Here the LD map shows the intensity of linkage disequilibrium of SNPs. In the map, the “flat”  curve means that the SNPs are in strong linkage disequilibrium, i.e., the recombination rarely occur between them, while the “steep” curve means the recombination occurs frequently in this part of chromosome. Samples are collected from the CEU (30 trios), CHB (45 unrelated individuals), JPT (45 unrelated individuals), YRI (30 both-parent-and-adult-child trios). There are 90 samples for CEU and YRI populations respectively, and 45 samples for each of CHB and JPT populations. Missing data are considered as a category in the calculation.

#### *Parkinson disease data*

The Parkinson disease SNPs data is based on a genome-wide genotyping of 270 individuals with idiopathic Parkinson Disease cases (case) and 271 neurologically normal controls (control) downloaded from the Coriell Institute for Medical Research (http://www.ncbi.nlm.nih.gov/sites/entrez?Db=gap). The genotyping was performed using the Illumina Infinium I and Infinium II assays. The Illumina Infinium I assay asseses 109,365 unique gene-centric SNPs while the Infinium II assay assesses 317,511 haplotype taggings SNPs based upon Phase I of the International HapMap Project. The Illumina Infinium I and II assays share 18,073 SNPs in common. Therefore, the combination of the two assays represents 408,803 unique SNPs. In the following experiment, SNPs with a > 1% missing percentage in all samples are not considered. After missing values are filtered out, the number of SNPs was decreased to 367440.

### Shrunken methodology

The nearest shrinkage centroid is developed to handle numerical microarray data sets. The main difference between gene expression and SNP data is that the expression values are continuous and SNPs are categorical [28].

In this paper, we make use of the shrinkage idea and apply the algorithm for categorical SNP data by using a genotype distribution measuring for categorical objects and modes instead of means for groups. These extensions will remove the numeric-only limitation of the nearest shrunken method and enable the classification process to be used to efficiently deal with genome-wide categorical SNP data sets.

 Let *x_ĳ_* be the categorical value for SNP *i* = 1, 2, …, *p* and samples *j* = 1, 2, …, *n*. There are *K* classes and let *C_k_* be indices of the *n_k_* samples in class *k*. The centroid of the *i *th SNP in class *k* is defined as:

	(1)

where mode is the category that with the highest appearance frequency.

The overall centroid for SNP *i* is:

	(2)

Let

	(3)

where  is the genotype distribution vector associated with *i* th SNP centroid in class *k*, and  is the genotype distribution vector associated with *i* th SNP overall centroid, ‖.‖_2_ is the Euclidean norm, s*_i_* is the pooled within-class standard deviation for SNP *i*:

	(4)

and

	(5)

*C_k_* denote the indices of the *n_k_* samples in class *k*, *s*_0_ is a positive constant included to prevent the possibility that a SNP with small deviation could produce a large *d_ĳ_.* In (3),we need to consider the distance from a class centroid to the overall centroid for the *i* th SNP. In our proposal, genotype distributions are used for measuring categorical SNPs data.

In the next step, the soft thresholding  can be defined similarly by:

	(6)

In (3),we can see that if the difference between a class centroid and the overall centroid is small, it demonstrates that the difference is insignificant or is just some noise in the classification process. Let t be a test sample, the class label of t is determined by:

	(7)

and

where *π_k_* is the prior probability of class *k*. It is the proportion of class *k* in the population. If it is unknown, it can be set to .

### Cross validation

A 10-fold cross validation is adopted in our classification procedure to evaluate the performance of the proposed nearest shrunken centroid method. In each trial, all the samples are randomly divided into 10 equal partitions. For each of the 10 partition groups, we select one of them as testing set and the remaining nine of them are considered as training sets. Ten trials are considered and the results are collected and based on this 10-fold cross validation procedure.

### SNP network construction

All the SNPs that selected by the shrunken metholodgy belong to 122 unique genes. We compute all the pair-wise functional similarities of these gene products using GOSemSim, a package of Bioconductor [29], which is an open source and open development software project for the analysis and comprehension of genomic data running in the platform of R. GOSemSim estimates the similarity scores of gene pairs according to their GO terms: molecular function (MF), biological process (BP) and cellular component (CC) [25]. In this paper, we only consider two of these terms: MF and BP and adopt Rel's method [30] to compute the similarity values, which is based on the information content of the GO terms and define information content as the frequency of each term occurs in the GO corpus. Afterwards, gene pairs that have a similarity value being greater than a threshold, were selected to construct several groups of genes using Cytoscape [31].

For the SNPs that involved in these groups of genes, we did a statistical analysis between these SNPs and all the other SNPs selected by our method using PLINK [24], which is a free, open-source whole genome association analysis toolset, designed to perform a range of basic, large-scale analysis in a computationally efficient manner. PLINK provides a logistic regression test for interaction that assumes an allelic model for both the main effects and the interactions. All pairwise combinations of SNPs can be tested. Odds ratio for interaction, *χ*^2^ statistic and asymptotic *P*-value will be provided in the output file. By constructing SNPs networks with SNP pairs that have *P* < 0.01 significance, we can figure out some potential SNP-SNP interactions that are still unknown.

## Results and discussion

### HapMap SNP data set

**Figure 1:**
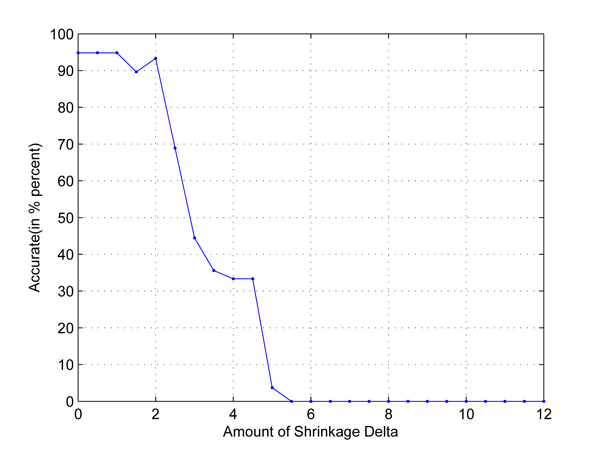
**CEU-CHB classification.** Two populations: CEU and CHB out of the 4 populations in HapMap data set are picked out to set up a two-class classification. The X axis is the amount of shrinkage Δ and Y axis is the accuracy (accuracy refers to the correctly classified samples in testing data sets in the 10-fold cross validation) obtained by using our shrunken method.

**Figure 2:**
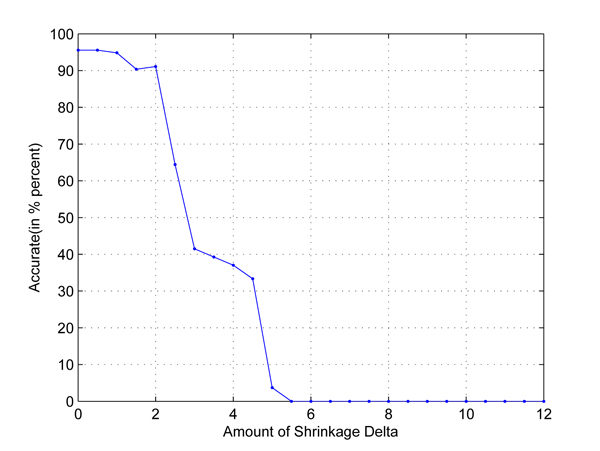
**CEU-JPT classification.** Two populations: CEU and JPT out of the 4 populations in HapMap data set are picked out to set up a two-class classification. The X axis is the amount of shrinkage Δ and Y axis is the accuracy (accuracy refers to the correctly classified samples in testing data sets in the 10-fold cross validation) obtained by using our shrunken method.

**Figure 3:**
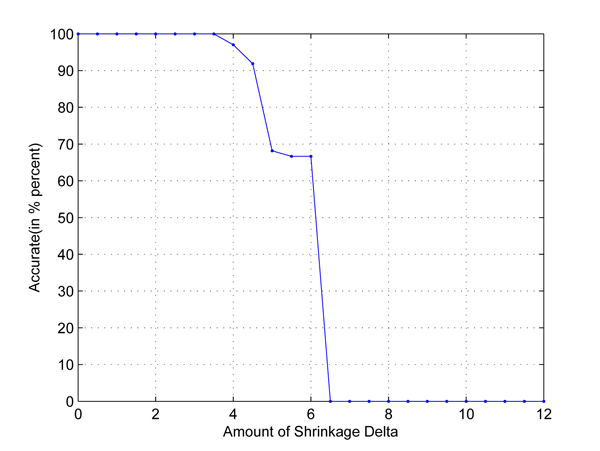
**YRI-CHB classification.** Two populations: YRI and CHB out of the 4 populations in HapMap data set are picked out to set up a two-class classification. The X axis is the amount of shrinkage Δ and Y axis is the accuracy (accuracy refers to the correctly classified samples in testing data sets in the 10-fold cross validation) obtained by using our shrunken method.

**Figure 4:**
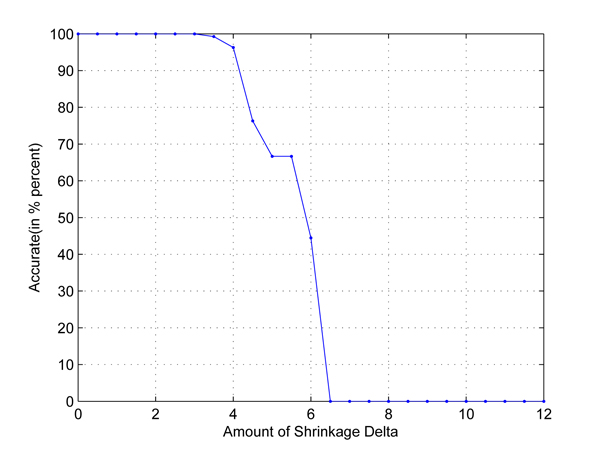
**YRI-JPT classification.** Two populations: YRI and JPT out of the 4 populations in HapMap data set are picked out to set up a two-class classification. The X axis is the amount of shrinkage Δ and Y axis is the accuracy (accuracy refers to the correctly classified samples in testing data sets in the 10-fold cross validation) obtained by using our shrunken method.

**Figure 5:**
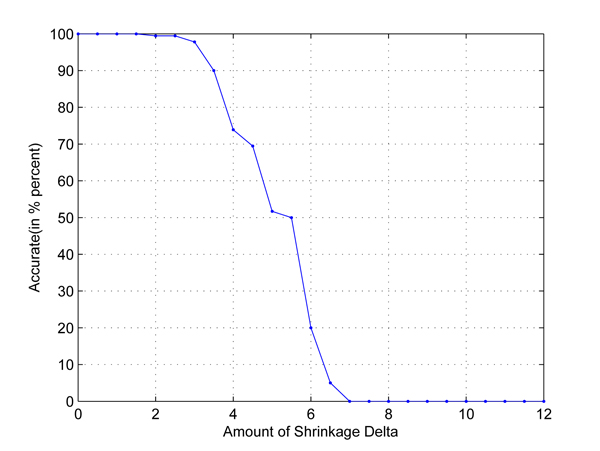
**CEU-YRI classification.** Two populations: CEU and YRI out of the 4 populations in HapMap data set are picked out to set up a two-class classification. The X axis is the amount of shrinkage Δ and Y axis is the accuracy (accuracy refers to the correctly classified samples in testing data sets in the 10-fold cross validation) obtained by using our shrunken method.

**Figure 6:**
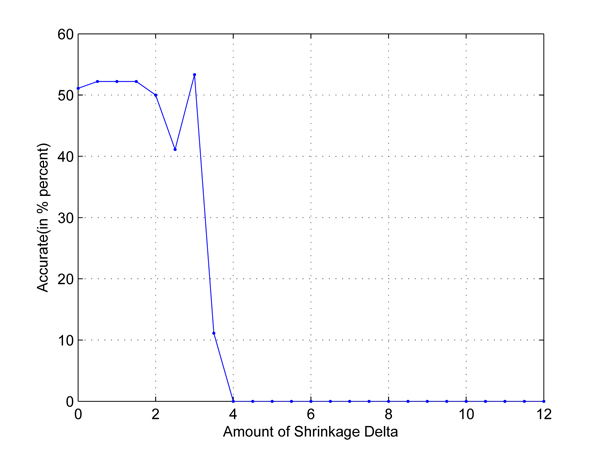
**CHB-JPT classification.** Two populations: CHB and JPT out of the 4 populations in HapMap data set are picked out to set up a two-class classification. The X axis is the amount of shrinkage Δ and Y axis is the accuracy (accuracy refers to the correctly classified samples in testing data sets in the 10-fold cross validation) obtained by using our shrunken method.

**Figure 7:**
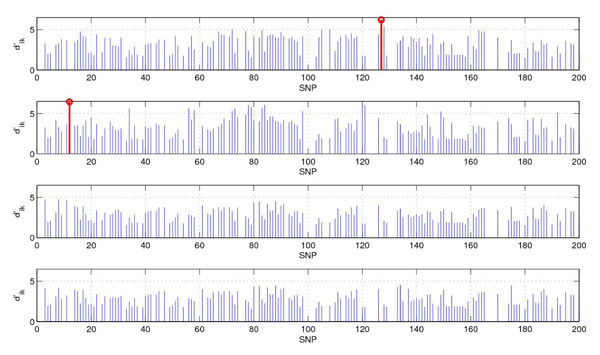
**The values of soft threshold.** The SNPs used in prediction and their values of  (from top to bottom are: CEU, YRI, CHB, JPT, Δ = 1.5). The values of  in blue in the figure mean that its corresponding SNP appears in all four populations, while the values of  in red represents its corresponding SNP shows in only one population.

In the first test, we take any two out of four populations in HapMap data set to set up two-class classification problems. Cross-validation is used to employ independent data sets. The results are shown in Figures 1, 2, 3, 4, 5, 6. As shown in these figures, we can see that all have a high accuracy of more than 90 percent, except the CHB-JPT classification problem, only about 50 percent, when the threshold Δ is less than 2. Then accuracy decreases as the amount of shrinkage increases since less SNPs are used in the prediction. The reason for the poor accuracy of CHB-JPT classification is that these two populations are quite similar on their SNPs, see Figure 7.

**Figure 8:**
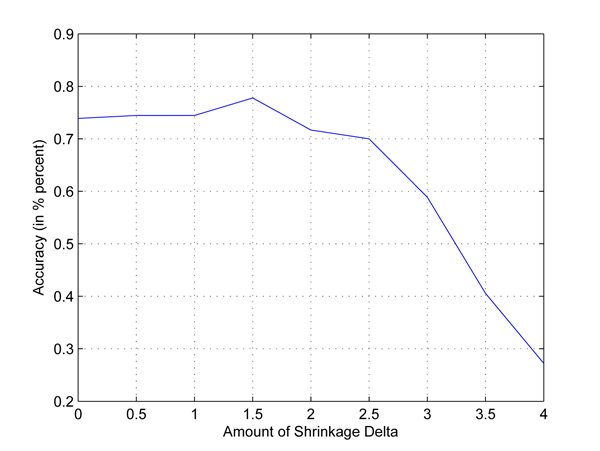
**Classification accuracy for four classes problem using 200 SNPs in Chromosome 22.** Four populations: CEU, CHB, YRI and JPT in HapMap data set are picked out to set up a four-class classification. The X axis is the amount of shrinkage Δ and Y axis is the accuracy (accuracy refers to the correctly classified samples in testing data sets in the 10-fold cross validation) obtained by using our shrunken method. Only 200 SNPs located in 3.44e7-3.5e7kb of chromosome 22 are used in this experiment.

**Figure 9:**
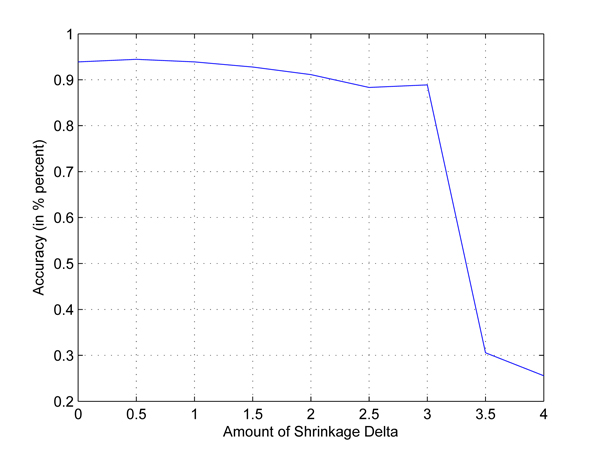
**Classification accuracy for four classes problem using all 51793 SNPs in Chromosome 22.** Four populations: CEU, CHB, YRI and JPT in HapMap data set are picked out to set up a four-class classification. The X axis is the amount of shrinkage Δ and Y axis is the accuracy (accuracy refer to the correctly classified samples in testing data sets in the 10-fold cross validation) obtained by using our shrunken method. All 51793 SNPs of chromosome 22 are used in this experiment.

In the second test, we consider a four-class classification problem, i.e., to classify the four populations: CEU, CHB, JPT and YRI. The setting is the same as that in the first experiment. Figure 8 shows the cross-validation classification accuracy using different values of Δ for 200 SNPs. The best accuracy is 77.78 percent when Δ = 1.5. When Δ < 1.5, there are a lot of SNPs to be used in the classification, but some of them are likely redundant. When Δ > 1.5, a lot of SNPs are not used, we may throw away some useful SNPs in the classification process. The confusion matrix in Table 1 shows that the prediction for CEU and YRI is quite good, but bad for CHB and JPT. In these two cases, the accuracy is not high. When we use all 51793 SNPs in chromosome 22 to perform the classification, the best accuracy is 94.44 percent (Δ = 0.5), see Figure 9.

**Table 1:** Confusion matrix when Δ = 1.5.

	CEU	YRI	CHB	JPT
**CEU**	43	0	1	1
**YRI**	0	45	0	0
**CHB**	0	0	30	15
**JPT**	0	0	23	22

Four populations: CEU, CHB, YRI and JPT in HapMap data set are picked out to set up a four-class classification. Only 200 SNPs located in 3.44e7-3.5e7kb of Chromosome 22 are used in this experiment. The confusion matrix is created when the best accuracy obtained under Δ = 1.5.

By shrinkage (Δ is set to 1.5), the number of SNPs used for classification is decreased from 200 to 143, 143, 142 and 142 for CEU, YRI, CHB, and JPT respectively. In Figure 7, we show the SNPs used in prediction and their value of . The values of  in blue in the figure mean that its corresponding SNP appears in all four populations, while the values of  in red represents its corresponding SNP shows in only one population. Next we show the centroid genotype distribution vector corresponding to the  in red in Table 2.

**Table 2:** Genotype distribution vector of 12th SNP (left) and 127th SNP (right).

	aa	aA	AA		aa	aA	AA
**CEU**	0	0.0667	0.9333	**CEU**	0.1556	0.4	0.3778
**YRI**	0.0667	0.5111	0.4222	**YRI**	0.0222	0.1333	0.8444
**CHB**	0	0.0444	0.9556	**CHB**	0	0	1
**JPT**	0	0.0222	0.9778	**JPT**	0	0	1

Illustration of genotype distribution vector of 12th SNP (left) and 127th SNP (right) in the four populations CEU, YRI, CHB and JPT in HapMap data set where A and a represent the major and minor alleles. Each distribution of the above three category (aa, aA, AA) is indicated by their distribution percentage in all samples.

As shown in Table 2, at 12th SNP, the genotype distribution vector of YRI is quite different from the others, similarly, at 127th SNP, the genotype distribution vector of CEU differs from those of the other three populations. The reason is that the mode of YRI is “aA”, while that of whole population is “AA”, and therefore YRI population has more variation and has a large value of .

### Parkinson disease SNPs data

Next we consider to use Parkinson disease data set to perform experiments to show the effectiveness of the shrunken methodology and construct SNPs networks. Table 3 shows the average classification accuracy results (correctly classified samples in testing data sets in the 10-fold cross validation) of all 22 chromosomes of Parkinson disease data set by using the nearest shrunken centroid program after 10-fold cross validation. We use the most frequent genotypes in case and control groups to be the modes for the program. The parameter Δ is tuned in each chromosome to obtain the highest accuracy in the test. To demonstrate the effectiveness of the proposed method, we also have a comparison with Park's [20] using the corresponding same data set. Here we use the numerical values (0,1,2,3) to represent different genotypes for Park's method. According to Table 3, the performance of our shrunken centroid method in terms of accuracy and numbers of selected SNPs is better than Park's method.

**Table 3:** Comparisons between the proposed method and Park's method.

		The Proposed Method			Park's Method		
Chromosome	Number of SNPs	Accuracy	Δ	Number of SNPs	Accuracy	Δ	Number of SNPs
1	29226	0.5926	0.893	21	0.5667	0.876	88
2	30298	0.6019	0.919	20	0.5722	0.965	87
3	25648	0.6056	0.880	26	0.5611	0.860	132
4	22315	0.6204	0.918	11	0.5945	1.024	42
5	22746	0.5796	0.926	22	0.5611	0.900	82
6	24334	0.6093	1.003	3	0.5204	0.944	49
7	19740	0.5797	0.901	7	0.5685	0.980	39
8	21384	0.5834	0.931	6	0.5741	0.979	35
9	18122	0.5426	0.932	7	0.5148	0.908	61
10	18525	0.6074	0.930	9	0.5593	0.999	47
11	18074	0.6352	0.964	7	0.5944	0.942	52
12	18186	0.6074	0.893	17	0.5667	0.975	31
13	13077	0.5870	0.906	9	0.5053	0.919	23
14	11728	0.5574	0.901	8	0.5259	0.962	26
15	10813	0.6037	0.905	8	0.5556	0.917	40
16	10892	0.5778	0.936	8	0.5611	0.946	37
17	10730	0.5815	0.919	4	0.5037	0.961	31
18	11677	0.5704	0.921	5	0.5071	0.956	27
19	7749	0.6037	0.887	7	0.5463	0.950	13
20	9647	0.6111	0.864	12	0.5907	0.951	33
21	6070	0.5833	0.875	8	0.4945	0.911	8
22	6459	0.6056	0.901	5	0.5444	0.924	23

**Average**		0.5930	0.914	10	0.5495	0.943	46

Illustration of the classification accuracies of all chromosomes of Parkinson disease genome-wide data set after a 10-fold cross validation and a detailed comparison between our proposed method and Park's method when filter out those SNPs whose missing percentage is >1%. The second column indicates the number of SNPs in each chromosome after filtering. Accuracy is the average accuracy after a 10-fold cross validation, Δ value and No of SNPs are obtained when the corresponding average accuracy is reached.

**Figure 10:**
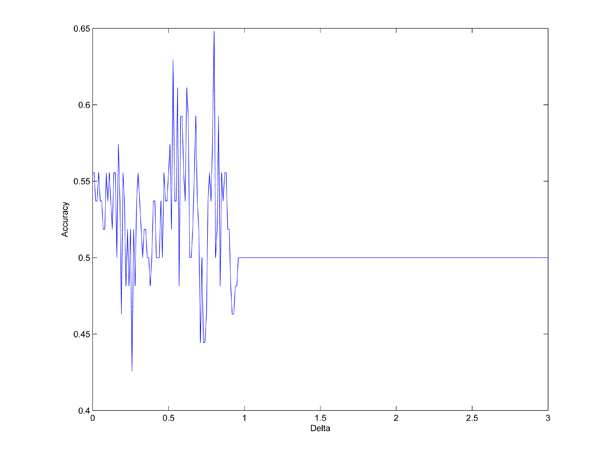
**Relationship between Δ and accuracy in Chromosome 14.** Illustration of the accuracy obtained in Chromosome 14 of Parkinson disease genome-wide data set when change Δ value from 0 to 3. For Chromosome 14, in each trial, all the 541 samples of both control and case are randomly divided into 10 equal partitions. For each of the 10 partition groups, we select one of them as testing set and the remaining nine of them are considered as training sets. 10 trials are considered and the results are collected based on this 10-fold cross validation procedure. This figure was drawn based on one of these ten trails when the highest accuracy (accuracy refers to the percentage of correctly classified samples over all test samples) is obtained. X axis refers to Δ value, it increases from 0 to 3. Y axis refers to the accuracy obtained in Chromosome 14 when using our method, it fluctuates when different Δ values are applied and the highest accuracy is obtained when Δ is equal to 0.8.

We also choose Chromosome 14 as an example to demonstrate the SNPs selected by the proposed method. Figure 10 shows the accuracies obtained when we increase Δ value from zero to three in one trial of the 10-fold cross validation. We can see from the figure that our method can get a reasonably good accuracy of 64.81% when Δ is equal to 0.8. By shrinkage, the number of SNPs selected for the classification is decreased from 11728 to 20. In Table 4, we show the genotype distributions of these 20 SNPs in the disease and control groups where A and a represent the major and minor alleles. The column under “Missing” refers to the missing percentages of genotypes in the groups. According to the table, we find that the SNP genotype distributions in two groups are quite different.

**Table 4:** Genotype distributions of selected 20 SNPs in Chromosome 14.

	Control Group				Disease Group			
SNPs	AA	Aa	aa	Missing	AA	Aa	aa	Missing
rs12434822	0.4391	0.4096	0.1513	0.0000	0.3000	0.5111	0.1889	0.0000
rs1952415	0.5055	0.4354	0.0591	0.0000	0.6185	0.2852	0.0963	0.0000
rs12050360	0.5424	0.3985	0.0591	0.0000	0.6519	0.2889	0.0592	0.0000
rs2248160	0.8155	0.1734	0.0111	0.0000	0.9000	0.1000	0.0000	0.0000
rs7146149	0.4760	0.4354	0.0812	0.0074	0.6174	0.3185	0.0704	0.0037
rs6573113	0.7232	0.2399	0.0369	0.0000	0.6037	0.3556	0.0370	0.0037
rs7560	0.6421	0.2878	0.0664	0.0037	0.5037	0.4000	0.0963	0.0000
rs1950902	0.6199	0.3506	0.0295	0.0000	0.7074	0.2519	0.0407	0.0000
rs11626809	0.8044	0.1882	0.0074	0.0000	0.7000	0.2889	0.0111	0.0000
rs1950764	0.7454	0.2398	0.0148	0.0000	0.6370	0.3445	0.0185	0.0000
rs11620883	0.8155	0.1808	0.0037	0.0000	0.9333	0.0630	0.0037	0.0000
rs3742837	0.6679	0.3026	0.0295	0.0000	0.5370	0.4222	0.0408	0.0000
rs8006322	0.3653	0.5388	0.0959	0.0000	0.4481	0.3889	0.1630	0.0000
rs12589063	0.3911	0.4428	0.1661	0.0000	0.2445	0.5370	0.2037	0.0148
rs7146193	0.3948	0.4465	0.1587	0.0000	0.2445	0.5481	0.2074	0.0000
rs8016079	0.7048	0.2731	0.0221	0.0000	0.8408	0.1481	0.0111	0.0000
rs12589195	0.6199	0.3284	0.0517	0.0000	0.5148	0.4482	0.0370	0.0000
rs7157079	0.8413	0.1550	0.0037	0.0000	0.9407	0.0593	0.0000	0.0000
rs11847484	0.7085	0.2767	0.0148	0.0000	0.8222	0.1704	0.0074	0.0000
rs1152781	0.5978	0.3136	0.0886	0.0000	0.4630	0.4555	0.0815	0.0000

Illustration of genotype distributions of selected 20 SNPs in Chromosome 14 of Parkinson disease genome-wide data set when filter out those SNPs whose missing percentage is >1%. The first column is the ID number of SNP. In Control/Disease group, major/major allele is represented by AA, major/minor is represented by Aa and minor/minor allele is represented by aa. Missing values under < 1% are also considered. Each distribution of the above four category is indicated by their distribution percentage in all samples.

We randomly select one trial of this 10-fold cross validation as an example to further analyze. In this trial, for all the 367440 SNPs from 22 chromosomes of Parkinson disease data set, there are totally 357 selected and 171 of them are located in gene coding area. Next we make use of the knowledge of these genes to construct SNPs networks. For the 122 genes that those 171 SNPs located in, we cluster the genes based on their similarity values using GOSemSim. The closely related biological process and molecular function roles of each gene were checked with GOSemSim with a threshold. When a similarity value between two genes is less than the threshold, their relationship is not considered. Therefore several groups of genes can be formed. As we are interested at gene-gene interactions, and we only consider the groups where the number of genes in these groups are more than one. In Table 5, we show the number of groups of genes formed by using different threshold values and the number of pairs of genes involved.

**Table 5:** Groups of genes formed for different threshold values.

GOSemSim thresholds	Number of Pairs of Genes		Number of Group of Genes	Number of SNPs Networks
0.18	499		1	10
0.19	448		3	9
0.20	137		9	6
0.21	114		9	6
0.22	111		10	6
0.23	106		10	6
0.24	100		10	6
0.25	78		12	6
0.26	70		10	5
0.27	46		10	2
0.28	27		10	2
0.29	26		9	2
0.30	22		8	2
0.31	22		8	2
0.32	22		8	2
0.33	22		8	2
0.34	17		8	2
0.35	13		7	1

Illustration of number of gene groups and number of gene pairs involved when using different GOSemSim thresholds. The first column is the GOSemSim thresholds from 0.18-0.35. The second column indicates the number of gene pairs involved in the gene network, i.e. the number of gene pairs whose similarity value is bigger than the GOSemSim threshold. The third column is the number of gene groups in the gene network when adopt a particular threshold. The last column indicates the number of SNPs networks constructing from the corresponding gene network under a *P* <0.01 significance.

**Figure 11:**
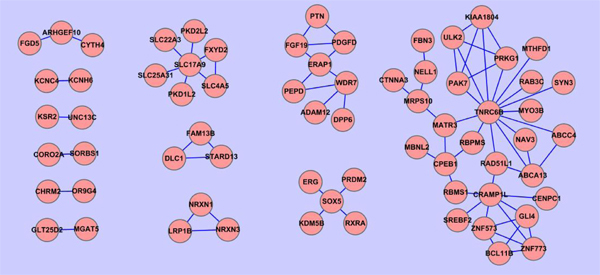
**Gene network when GOSemSim threshold=0.25.** Gene network constructed using Cytoscape. Gene pairs are computed the similarity values using GOSemSim and gene pairs that have a > 0.25 threshold are grouped together. Every node in the figure is labeled as its gene symbol and the edge between two genes indicates whether this pair of genes has a > 0.25 threshold or not. There are 12 clusters in the gene network when threshold=0.25.

We see in Table 5 that the number of groups of genes increases when the threshold value increases as more groups are formed. However, when threshold value further increases, the number of groups is reduced as each group just contains one gene. According to Table 5, we select the threshold to be 0.25 for analysis as the number of groups of genes is higher than those using the other threshold values. Figure 11 demonstrates the group of genes constructed by our method when threshold is equal to 0.25. Gene pairs that are grouped in the same group suggest a strong potential for interaction effects in biological process. We can see from this figure that there are 12 groups, including 68 genes.

**Figure 12:**
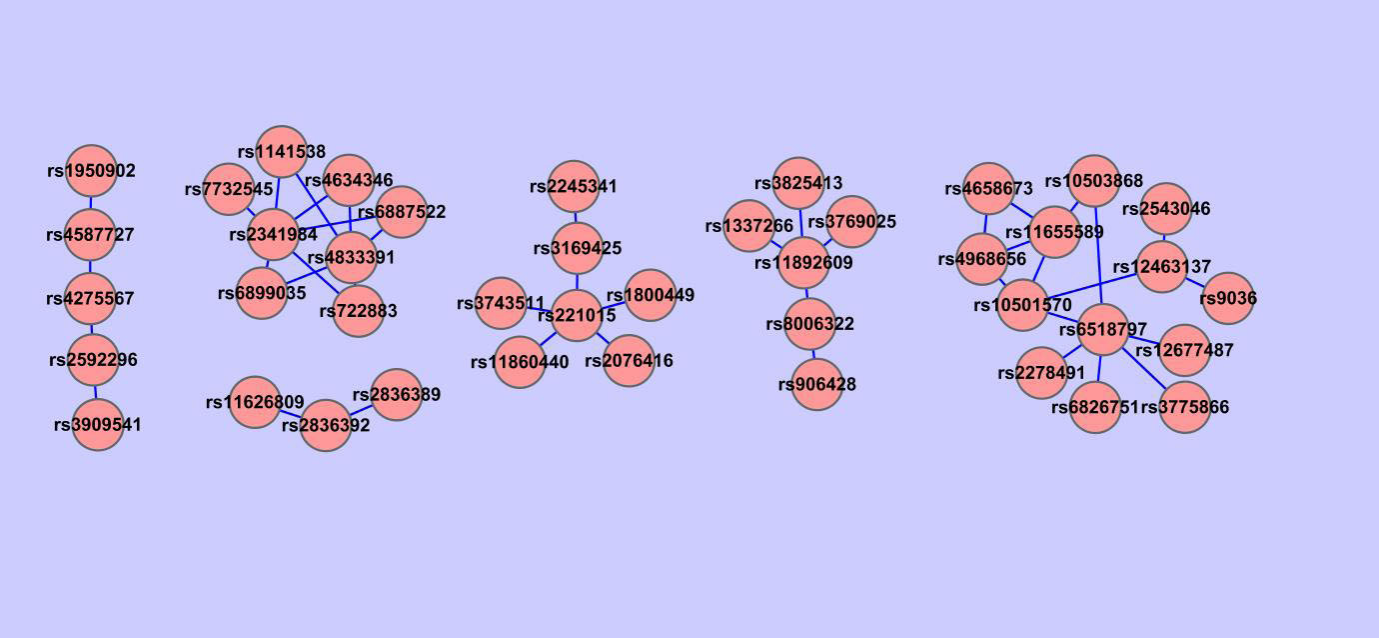
**SNPs network when GOSemSim threshold=0.22.** SNPs network constructed using Cytoscape. For each group of the gene network where gene pairs have > 0.22 similarity value, all the pairwise SNP-SNP interactions are checked using PLINK between SNPs involved in the groups of genes and all the other SNPs selected by the shrunken method. SNPs network is constructed based on the *P* value of PLINK epistasis test. Each node in the figure is labeled as its SNP ID and the edge between two SNPs indicates whether this pair of SNPs are interacted under a *P* < 0.01 significance. There are 6 SNPs networks in this figure.

**Figure 13:**
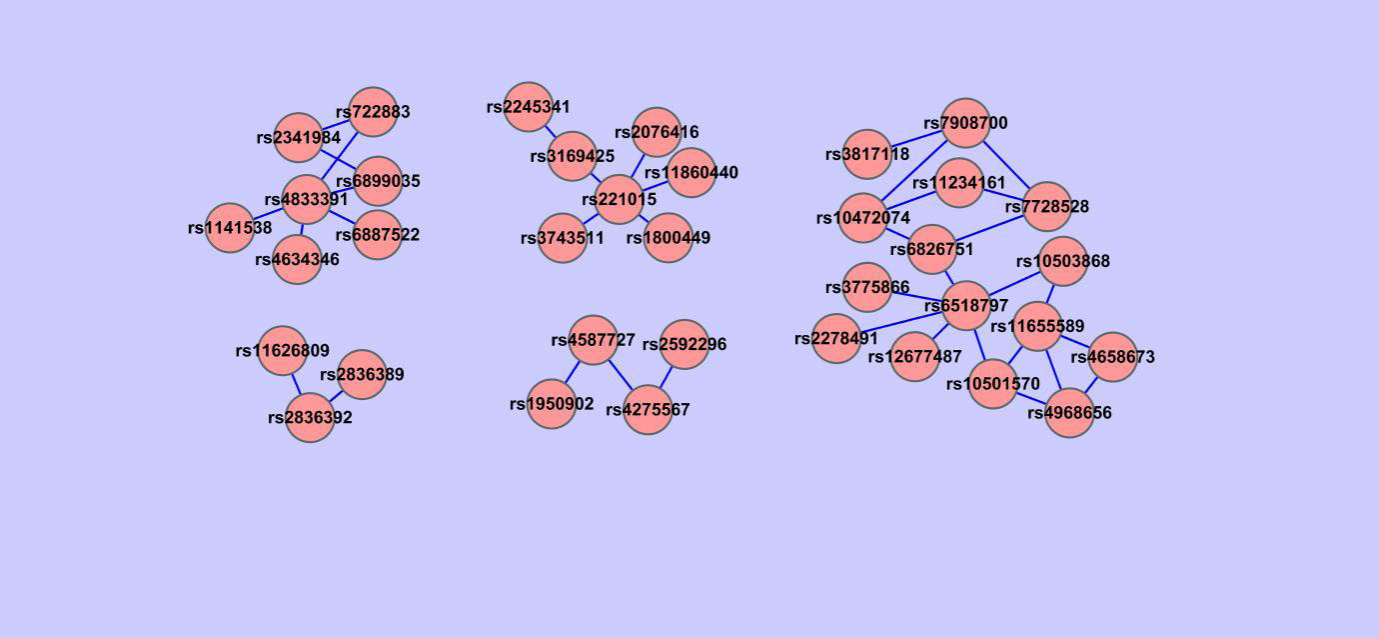
**SNPs network when GOSemSim threshold=0.26.** SNPs network constructed using Cytoscape. For each group of the gene network where gene pairs have › 0.26 similarity value, all the pairwise SNP-SNP interactions are checked using PLINK between SNPs involved in the groups of genes and all the other SNPs selected by the shrunken method. SNPs network is constructed based on the *P* value of PLINK epistasis test. Each node in the figure is labeled as its SNP ID and the edge between two SNPs indicates whether this pair of SNPs are interacted under a *P* < 0.01 significance. There are 5 SNPs networks in this figure.

**Figure 14:**
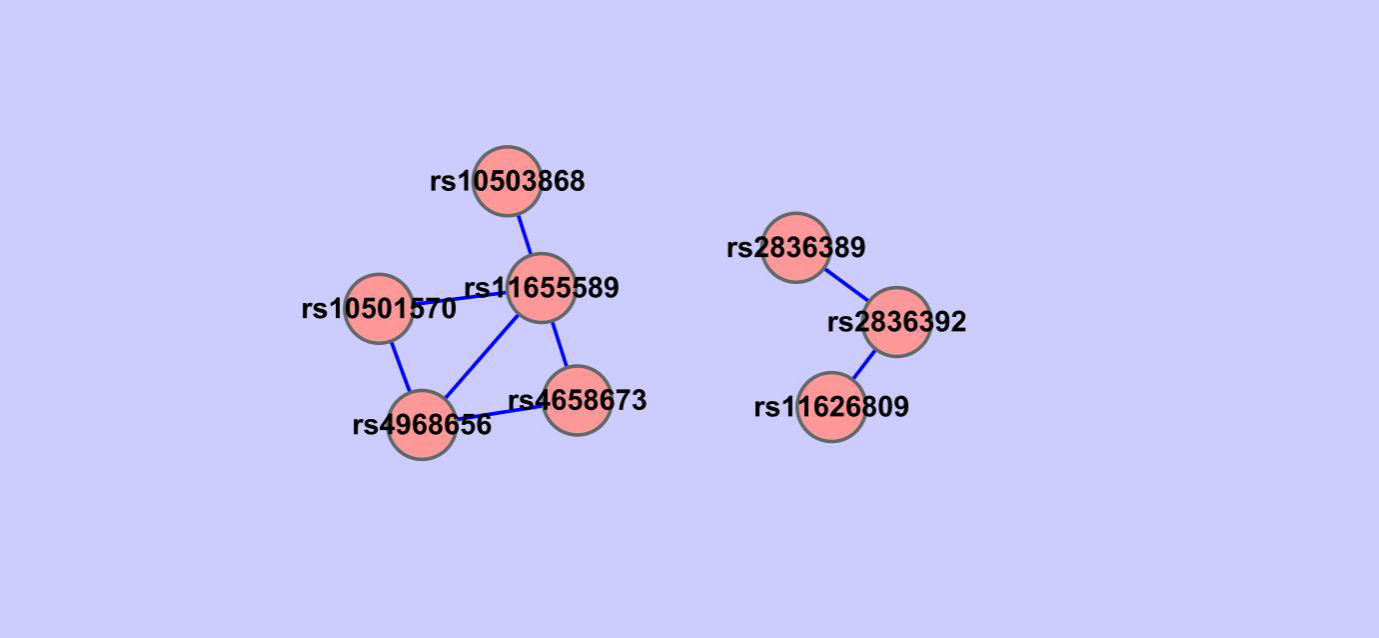
**SNPs network when GOSemSim threshold=0.27.** SNPs network constructed using Cytoscape. For each group of the gene network where gene pairs have > 0.27 similarity value, all the pairwise SNP-SNP interactions are checked using PLINK between SNPs involved in the groups of genes and all the other SNPs selected by the shrunken method. SNPs network is constructed based on the *P* value of PLINK epistasis test. Each node in the figure is labeled as its SNP ID and the edge between two SNPs indicates whether this pair of SNPs are interacted under a *P* < 0.01 significance. There are 2 SNPs networks in this figure.

For each group of genes constructed, we check all the pairwise SNP-SNP interactions using PLINK between SNPs involved in the group of genes and all the other SNPs selected by the shrunken method. Based on the *P*-value of PLINK epistasis test, we construct SNPs networks. Because there are more groups of genes when the threshold value in GOSemSim is in between 0.22-0.28, we are interested in their corresponding SNPs networks. In particular, we show in Figures 12, 13, 14 that SNPs networks when the threshold values are 0.22, 0.26 and 0.27 respectively. We find that there are two SNPs networks as shown in Figure 14 appearing frequently among the networks constructed when the threshold value in GOSemSim is in between 0.22-0.28. Table. 6 shows all SNP pairs of these interesting SNPs networks that have *P* < 0.01 significance interactions in Figure 14.

**Table 6:** Pair-wise interactions among SNPs when GOSemSim threshold=0.27 (*P* <0.01).

Chromosome1	Chromosome2	Gene1	Gene2	SNP1	SNP2	OR_int	*Χ* ^2^	*P* -value
1	17	intergenic	KCNH6	rs4658673	rs4968656	0.4873	10.52	0.001183
17	17	intergenic	KCNH6	rs11655589	rs4968656	0.5365	10.43	0.001242
11	17	intergenic	intergenic	rs10501570	rs11655589	0.5096	8.76	0.003083
21	21	ERG	ERG	rs2836389	rs2836392	0.5642	7.55	0.006017
8	17	intergenic	intergenic	rs10503868	rs11655589	0.5543	7.49	0.006223
14	21	RAD51L1	ERG	rs11626809	rs2836392	2.4410	7.40	0.00652
1	17	intergenic	intergenic	rs4658673	rs11655589	0.5534	7.37	0.00665
11	17	intergenic	KCNH6	rs10501570	rs4968656	0.5551	6.66	0.009844

Pair-wise interactions among SNPs when GOSemSim threshold=0.27 with *P* <0.01 significance. For each group of the gene network where gene pairs have > 0.27 similarity value, all the pairwise SNP-SNP interactions are checked using PLINK between SNPs involved in the groups of genes and all the other SNPs selected by the shrunken method. SNPs network is constructed based on the *P* <0.01 value of PLINK epistasis test. This table shows the detailed results of PLINK where the first two columns are the chromosomes the SNPs located in, the third and forth columns are the corresponding gene symbol of the SNPs, the fifth and sixth columns are the SNP ID and the last three columns are the odds ratio for interaction, *χ*^2^ test and *P* value in the PLINK test.

We find some interesting relationships from these two SNPs networks. For example, for SNPs rs11626809 and rs2836392, which are highly interacted, their corresponding genes are RAD51L1 and ERG respectively, but located in different clusters in gene network, which means that maybe we can merge these two clusters in gene network together. Another example, rs4968656 is interacted with rs4658673, which is located in intergenic area and do not have a record in Gene Ontology until now, maybe we can make use of rs4968656's gene information, KCNH6, to further analyze the inner functions of rs4658673 and extend GO afterward.

Indeed some of the SNPs selected by the shrunken method are directly or indirectly related to PD. For example, ERG and anatomical abnormalities are reported to cause retinopathy in dementia with Lewy bodies [32], which share similar symptoms with PD and are thought to be related to PD, or that they sometimes happen together. KCNH6, located in Chromosome 17, are reported to have diverse functions include regulating neurotransmitter release, heart rate, insulin secretion, neuronal excitability, epithelial electrolyte transport, smooth muscle contraction, and cell volume. These characteristics are also the symptoms of PD.

## Conclusions

In this paper, we review the method of nearest shrunken centroid for gene expression data, and extend it to tackle SNP data classification. The main contribution of this paper is to develop a shrunken dissimilarity measure to handle SNP data classification problems. The method can be implemented on a PC very efficiently. The relevant SNPs are selected for HapMap data and Parkinson disease data. Experimental results are also reported to show the effectiveness of the method. In particular, we find some SNPs that contain in some genes which is relevant to Parkinson disease. Based on the SNPs network, we can have some unknown relationships between their corresponding genes, which can be considered as an extension of existing GO knowledge. In the future, detailed biological analysis of SNPs of other genome-wide SNP data sets will be studied. The genomic variation of data sets can take account of functional as well as linkage disequilibrium information. More importance is attached to some SNPs than others, based on their positions within the coding or regulatory regions or splice sites.

## Competing interests

The authors declare that they have no competing interests. 

## Authors contributions

MN designed this study and developed the new algorithm. YL designed this study, coded the program, and ran the experiments. Two authors wrote the manuscript.
